# Nanoemulsified *D*-Limonene Reduces the Heat Resistance of *Salmonella* Senftenberg over 50 Times

**DOI:** 10.3390/nano7030065

**Published:** 2017-03-15

**Authors:** María Ros-Chumillas, Alberto Garre, Javier Maté, Alfredo Palop, Paula M. Periago

**Affiliations:** 1 Food and Agricultural Engineering Department, Regional Campus of International Excellence “Campus Mare Nostrum” School of Agricultural Engineering, Technical University of Cartagena, Paseo Alfonso XIII 48, 30203 Cartagena, Spain; may.ros@upct.es (M.R.-C.); alberto.garre@upct.es (A.G.); j_ms_86@hotmail.com (J.M.); paula.periago@upct.es (P.M.P.); 2 Institute of Plant Biotechnology, Regional Campus of International Excellence “Campus Mare Nostrum”, Technical University of Cartagena, 30202 Cartagena, Spain

**Keywords:** food, nanomaterials, safety, nanoemulsions, *D*-limonene, heat resistance, *Salmonella* Senftenberg, antimicrobial, combined effect

## Abstract

*Salmonella* Senftenberg is a pathogen agent causative of foodborne disease and it is considered the most heat-resistant serovar within this genus. Food industries use heat treatment and chemical antimicrobials in order to eliminate this microorganism in food, but consumers prefer natural antimicrobials as essential oils and their components. This study evaluates the combined effect of thermal treatments and different concentrations of *D*-limonene nanoemulsion on the inactivation of *Salmonella* (*S.*) Senftenberg. The results showed an important effect of the nanoemulsified *D*-limonene on the heat resistance of *S.* Senftenberg. The δ_50 °C_ value was reduced by 85%, 96% and 98% when 0.1, 0.5 and 1 mM of nanoemulsified *D*-limonene was added to the heating medium. The effect was kept along all the heating temperatures researched and the shape of the survival curves did not change with the addition of the antimicrobial. The results obtained in this research could be very useful for food industries for optimizing or improving heat treatments applied to food.

## 1. Introduction

Consumers’ demand for high-quality, minimally processed or fresh food has increased in developed countries. While consumption of these kinds of foods has increased, the incidence of foodborne illnesses linked to them has grown as well. One of the most important foodborne pathogens is *Salmonella* spp., which can cause gastroenteritis infection. In fact, there are 94 million cases of gastroenteritis and 155,000 deaths globally because of *Salmonella* each year, and nearly 85% of these cases are directly related to food [1]. *Salmonella* Senftenberg is one of the serovars causing salmonellosis, and it is isolated from a range of different foods (seafood, eggs, etc.) [2,3,4]. It is considered one of the most heat-resistant serovars within the *Salmonella* genus [5,6].

Heat treatment is the most used method in the food industry to eliminate pathogenic microorganisms from food products. However, traditional pasteurization does not provide a sufficient log cycle reduction in *S.* Senftenberg [5,7] due to its high heat resistance, so this serovar has a relevant technological interest. Another method used to reduce microbial contamination in food is chemical preservation. The addition of preservatives in foods has been used for centuries. For instance, weak acid preservatives such as sorbic, benzoic and acetic acids are used in fruit juices, vegetables, beverages and sauces to avoid yeasts, moulds and bacteria [8,9]. Nevertheless, consumers prefer natural preservatives or antimicrobials such as essential oils and their components.

Essential oils are natural products extracted from plant material (flowers, buds, herbs, roots, leaves or fruits), and their use as natural preservatives in many foods is gaining interest because of their antibacterial, antifungal, antioxidant and anti-carcinogenic properties [10,11]. Some studies have demonstrated the antibacterial activity of essential oils against pathogenic bacteria, such as *Escherichia coli*, *Listeria monocytogenes* and *Salmonella* Typhimurium [12,13,14,15]. The major active components of essential oils are terpenes, phenols, and aldehydes. These components act against the cell cytoplasmic membrane, affecting the unsaturated fatty acid on the bacterial membrane and thus altering its structure. In fact, terpene compounds, such as limonene, cause the loss of membrane integrity and dissipation of the proton-motive force [14,16]. *D*-limonene is an important flavor component in citrus essential oil (e.g., lemon, lime, orange, mandarin, etc.), generally regarded as safe (GRAS) [17].

The combination of heat treatment and essential oils and their components has been extensively researched and even used to reduce microbial contamination in food, mainly because of their interesting synergistic effects [18,19,20,21]. However, essential oils have technological limitations caused by their hydrophobic bioactive molecules and their organoleptic properties [17]. In order to improve the limitation of hydrophobicity, nanoemulsion technology has emerged and has been proved to solve the problem of immiscibility in aqueous media [15,22,23,24]. The subcellular size of nanoemulsion droplets provides physical stability of the encapsulated active substances and increases the distribution of antimicrobial agents in food [25] and other matrices, such as textiles [26]. Nanometric-range emulsion droplets fuse with the bacterial cell membrane, destabilizing and disrupting it, resulting in the leakage of critical intracellular constituents [27,28]. Nanoemulsions have shown promising results when used to deliver antimicrobial compounds of essential oils, since nanoemulsified essential oil compounds show similar or even improved antimicrobial effects compared to the same substances added directly [14,22,29,30,31]. However, only Maté et al. [32,33] have studied the synergistic effect of a mild heat treatment and an essential oil nanoemulsion. The synergistic effect led to a 100-fold reduction of the thermal resistance of *L. monocytogenes* both in culture medium [32] and in apple juice [33], which is by far the largest reduction in the heat resistance of a microorganism ever published when combining heat with natural antimicrobials.

Gram-positive bacteria, such as *L. monocytogenes*, are usually more sensitive to antimicrobials than Gram-negative bacteria, because of the nature of their cell walls. Then, the possibility exists that this dramatic synergistic effect could be reduced or even completely lost when trying it in a Gram-negative bacterium. For this reason, the aim of this study was to evaluate the effect of a combined treatment of heat with different concentrations of nanoemulsion of *D*-limonene on the inactivation of *S*. Senftenberg in tryptic soy broth (TSB).

## 2. Results

**Figure 1:**
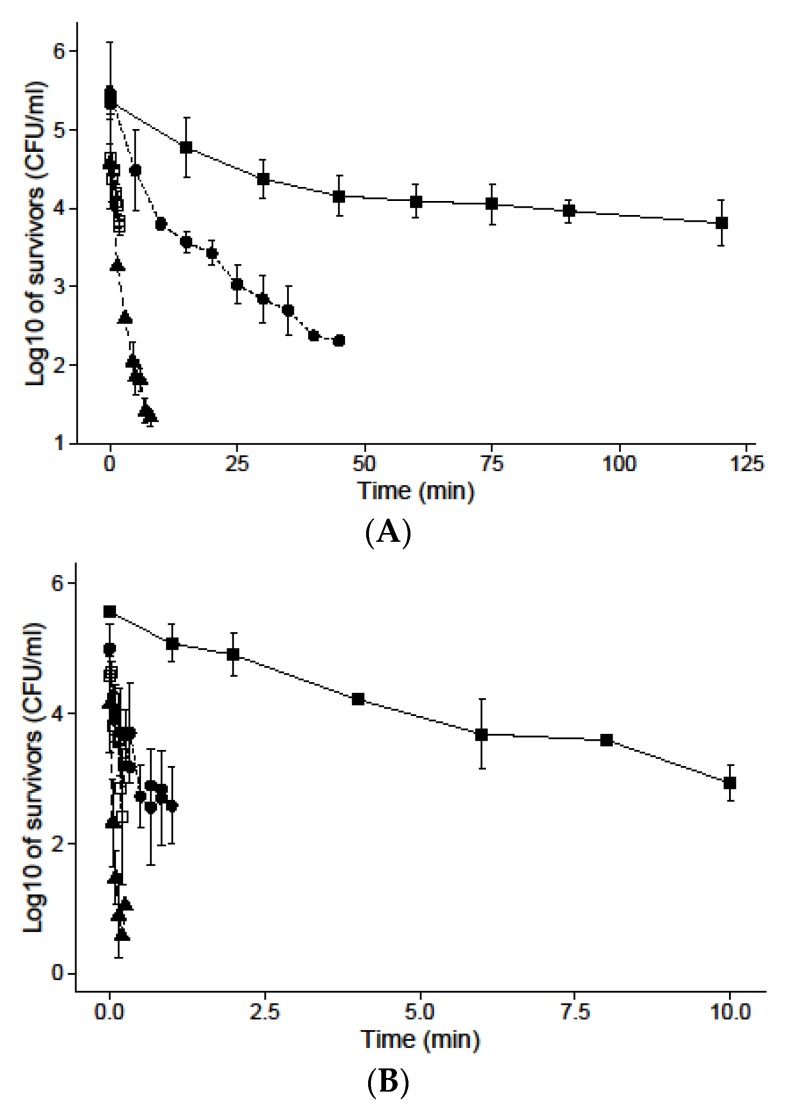
Survival curves of *S.* Senftenberg in Tryptic Soy Broth (TSB) at 50 °C (**A**) and 55 °C (**B**) with 0 (closed squares), 0.1 (circles), 0.5 (open squares) and 1 (triangles) mM *D*-limonene nanoemulsified.

Figure 1 shows the survivor curves observed when *S*. Senftenberg was exposed to different concentrations of *D*-limonene nanoemulsified at 50 °C (Figure 1A) and 55 °C (Figure 1B). At 50 °C, the reduction in the survivors of the control (without *D*-limonene nanoemulsion) reached one-and-a-half log cycles in approximately 125 min. An important decrease of the thermal resistance of *S*. Senftenberg was observed when the concentration of *D*-limonene increased. When nanoemulsified *D*-limonene at a concentration of 0.1 mM was added, the number of survivors was reduced three log cycles in 48 min and the same level of reduction was reached in less than 5 min when 0.5 and 1 mM of *D*-limonene were used (Figure 1A). Similar decreases of the thermal resistance when increasing the concentration of *D*-limonene were also observed at 55 °C (Figure 1B).

Table 1 summarizes the statistical information of the model fit for the whole set of curves fitted. The low values of the standard deviations of the model parameters estimated (lower than 10% of the estimated value) and the low Root Mean Squared Error (RMSE) obtained for every case, together with a visual inspection of the fitted curves, indicate that the fitted models have succeeded in describing the temporal variation of the bacteria concentration.

The impact that nanoemulsified *D*-limonene has on the thermal resistance of *S*. Senftenberg is reflected in the variations of the values estimated for parameter $\delta_{50\;{}^{\circ}C}$. The addition of 0.1 mM of *D*-limonene nanoemulsion caused an 85% reduction of the $\delta_{50\;{}^{\circ}C}\;$ parameter with respect to the control samples, whereas the 0.5 mM and 1 mM concentrations resulted in further reductions of 96% and 98%, respectively. When trying to find a correlation between the $\delta_{50\;{}^{\circ}C}$ values (or the log $\delta_{50\;{}^{\circ}C}$ values) with the *D*-limonene concentrations, neither an exponential nor lineal correlation was found. This means that the addition of just the lowest concentration of *D*-limonene tried leads to a dramatic decrease in the thermal resistance of the microorganism. Further increments of the concentration of *D*-limonene only lead to relatively minor reductions in the heat resistance of the microorganism.

On the other hand, the *D*-limonene nanoemulsion has no clear effect on the *z* value. The *z* values ranged from 3.14 °C to 3.83 °C (Table 1) and, although significant differences were found between some of the values, there was no clear trend with the increasing *D*-limonene concentrations. Hence, the effect the addition of nanoemulsified *D*-limonene has on the heat resistance of *S*. Senftenberg was somehow kept along all the heating temperatures researched.

## 3. Discussion

Our results showed a dramatic decrease of the thermal resistance of *S*. Senftenberg when nanoemulsified *D*-limonene was added to the heating medium. This reduction of about 50 times when a concentration of 1 mM nanoemulsified *D*-limonene was added can be regarded as among the biggest published in the literature when natural antimicrobials and heat are combined. Only Luis-Villarroya et al. [34] published a decrease of more than 40 times in the time to achieve a five log reduction in the population of *E. coli* O157:H7 when adding a propolis-based dietary supplement to pH 4 buffer. Also, Maté et al. [32] observed a reduction of about 100 times in the heat resistance of *L. monocytogenes* when adding 0.5 mM nanoemulsified *D*-limonene to the TSB broth used as a heating medium. In this case, the thermal resistance of *S*. Senftenberg was “only” reduced about 25 times when adding the same concentration of nanoemulsified *D*-limonene (from $\delta_{50\;{}^{\circ}C}$ = 32.13 min with no *D*-limonene to $\delta_{50\;{}^{\circ}C}$ = 1.38 min with 0.5 mM *D*-limonene; Table 1) and it was necessary to double the concentration of nanoemulsified *D*-limonene (to 1 mM) to reduce the thermal resistance about 50 times (to $\delta_{50\;{}^{\circ}C}$ = 0.65 min; Table 1). In spite of the reduction in the heat resistance of *S*. Senftenberg being smaller than that previously found for *L. monocytogenes*, it can be regarded as among the highest reductions caused by a combined process with heat and an antimicrobial ever found.

The fact that the effect was kept along all the heating temperatures tested agrees with results previously published by Maté et al. [32] with *L. monocytogenes*. Maté et al. [32] hypothesized that the dramatic decrease of the thermal resistance of *L. monocytogenes* was caused by the use of the *D*-limonene in the form of a nanoemulsion, which has been shown to improve the solubility and distribution of oily antimicrobials in aqueous media [35]. As a consequence, the antimicrobial can reach the cell more easily. Maté et al. [33] also found that the same effect was shown in a food product, such as apple juice. This finding was also very relevant, since most of the effect found with the combination of heat plus antimicrobials in culture medium is lost when they are applied in food products [34,36]. Even Maté et al. [33] found that this dramatic effect could be almost completely lost depending on the composition of the food, showing that in carrot juice containing fat and fiber, the effect was lacking, but when these components were removed from the carrot juice, the effect was, again, dramatic.

Food processors could take advantage of this effect, since adding a small amount of this antimicrobial, in the form of a nanoemulsion, could help them to considerably reduce the severity of the thermal treatment applied to processed foods.

## 4. Materials and Methods

### 4.1. Bacterial Strain

*S*. Senftenberg CECT 4565 used in this study was supplied by the Spanish Type Culture Collection (CECT, Valencia, Spain).

During this investigation it was maintained on slants of Tryptic Soy Agar (TSA) (Scharlau Chemie, Barcelona, Spain) at 4 °C until use. The culture was grown overnight (approx. 20 h) at 37 °C in Tryptic Soy Broth (TSB) (Scharlau Chemie, Barcelona, Spain) until the stationary phase of growth was reached.

### 4.2. Preparation of Nanoemulsions

The nanoemulsions of *D*-limonene (Sigma Aldrich Chemie, Steinheim, Germany) were prepared following the protocol described by Maté et al. [30] and based on catastrophic phase inversion method [37]. Briefly, aqueous phase was prepared by mixing 55 mL of sterile distilled water and 27.5 mL of propylene glycol (Panreac, Barcelona, Spain). To elaborate the oily phase, 6 mL of Tween 80 (Panreac, Barcelona, Spain) were mixed with 11.5 mL of *D*-limonene. The nanoemulsion was prepared by slowly adding aqueous phase into the oily phase with gentle agitation at room temperature. The addition rate of aqueous phase was kept constant at approximately 1.0 mL/min with continuous stirring. A water-in-oil emulsion with a high oil-to-water ratio was formed, and then increasing amounts of water were added to the system, until a phase inversion occurred and an oil-in-water emulsion was formed, with continuous stirring for 6 h. Final concentration of *D*-limonene in the nanoemulsion was 1 M. All the ingredients in the nanoemulsion (*D*-limonene, propylene glycol and Tween 80) are considered as GRAS substance and permitted as food additives in the European Union.

Nanoemulsions were aliquoted in pre-sterilized test tubes and stored in refrigeration until use. Droplet size was determined at the beginning and at the end of the experiment. Size distribution of the oil droplets were determined by the laser light scattering method using Mastersizer 2000 (Malvern Instruments, Worcestershire, UK), as already described [30]. No differences were found in size distribution along the time the present research was performed. Previous experiments [30] had shown a stability of the nanoemulsion over a six month period of time without significant modification of the droplet size or phase separation.

### 4.3. Heat Treatments

Thermal inactivation kinetics for *S*. Senftenberg in TSB supplemented with 0.1, 0.5 and 1 mM *D*-limonene were determined at different constant temperatures in a thermoresistometer Mastia [38]. *D*-limonene was added to pre-sterilized TSB in sterile conditions. Then, the vessel of the thermoresistometer was filled with 400 mL of pre-sterilized TSB supplemented with *D*-limonene nanoemulsion.

Heat treatments were conducted at 50.0, 52.5 and 55.0 °C. Once the heating medium temperature had attained stability (±0.05 °C), it was inoculated with 0.2 mL of the cell culture (approx. 10^8^ cells·mL^−1^). At preset intervals, 1 mL samples were collected into sterile test tubes, which were kept in ice until decimal dilutions were performed. Surviving cells were enumerated in tryptic soy agar (TSA, Scharlau Chemie, Barcelona, Spain). Plates were incubated for 24 h at 37 °C. Each treatment was assayed by triplicate in independent experiments performed in different days.

### 4.4. Mathematical Modelling and Data Analysis

The isothermal inactivation of *S*. Senftenberg has been described using the model proposed by Mafart et al. [39], due to the strong tail effect observed in the experimental data. This model assumes that the time that an individual microorganism in the microbial population can withstand a constant thermal stress follows a Weibull probability distribution. Hence, the fraction of survivors (*S*) varies through time (*t*) as described in Equation (1):

(1) $$\log_{10}S\left(t\right)=-{\left(\frac{t}{\delta \left(T\right)}\right)}^{p},$$

where *p* and δ(*T*) are the shape and scale parameters of the Weibull distribution, respectively. The shape parameter defines the curvature of the survivor curves. Values of p greater than one generate concave lines, while values lower than one result in convex curves. In case p equals 1, the survivor curves are log-linear. This parameter is considered temperature independent.

From the point of view of the microbiology, the scale parameter, δ(*T*), provides the time required for the first logarithmic reduction in the fraction of survivors. It is considered that this parameter follows an exponential relationship with temperature (T) according to Equation (2). The reference temperature ($T_{\mathrm{ref}}$) has been set to 50 °C for the analysis.

(2) $$\log_{10}\delta \left(T\right)=\log_{10}\delta_{\mathrm{ref}}+\frac{T_{\mathrm{ref}}-T}{z},$$

The sensitivity of the microbial population to temperature variations is provided by the parameter *z*. This parameter is defined as the temperature increase required for a 10-fold reduction of δ(*T*).

The model fitting has been performed using the bioinactivation package of *R* [40], which includes functions for parameter estimation from isothermal inactivation experiments using a one-step non-linear regression algorithm. The goodness of the fit has been assessed using the Root Mean Squared Error (RMSE) of the prediction generated by the adjusted model (Equation (3)).

(3) $$\mathrm{RMSE}=\sqrt{\frac{1}{n}\sum^{\text{​}}{\left(\log_{10}S-log_{10}\hat{S}\right)}^{2}},$$

Preliminary analyses have shown that the addition of *D*-limonene had a reduced impact on the value of parameter *p* of the Weibull model. Hence, the model fitting has been performed in two steps. Firstly, the model has been calibrated fitting the values of both δ and *p* individually for the results obtained for each *D*-limonene concentration (control, 0.1, 0.5 and 1 mM). On a second step, the model fitting has been repeated fixing the value of *p* to the mean of the values obtained in the previous step (p=0.52).

## 5. Conclusions

The addition of the *D*-limonene nanoemulsion to the TSB dramatically decreased the resistance of *S*. Senftenberg to isothermal stresses for all the temperatures studied.

This research expands the findings of the tremendous potential of nanoemulsified oily antimicrobials when combined with thermal treatments, formerly shown on *L. monocytogenes* [32,33], to Gram-negative bacteria, which are well known to show increased resistance to antimicrobials in comparison to Gram-positive bacteria. This result opens possible uses in the food industry.

## Figures and Tables

**Table 1:** Summary of the model fit to the survival curves of *S.* Senftenberg in Tryptic Soy Broth (TSB). Estimated values and standard deviations of the parameters of the Mafart model, as well as the Root Mean Squared Error (RMSE) of the fitted curves. The value of *p* was fixed to 0.52 for every model calibration.

*D*-Limonene (mM)	$\delta_{50\;{}^{\circ}C}\;\;$ (min)	*z* (°C)	*p*	RMSE
Control (0)	32.13 ± 3.12	3.83 ± 0.18	0.52	0.41
0.1	4.71 ± 0.30	3.33 ± 0.10		0.38
0.5	1.38 ± 0.09	3.65 ± 0.12		0.40
1	0.65 ± 0.03	3.14 ± 0.06		0.40
